# Application of a core genome sequence typing (cgMLST) pipeline for surveillance of *Clostridioides difficile* in China

**DOI:** 10.3389/fcimb.2023.1109153

**Published:** 2023-03-13

**Authors:** Yuan Yuan Wang, Lu Xie, Wen Zhu Zhang, Xiao li Du, Wen Ge Li, Lu Lu Bia, Zhi Gang Cui, Yuan Wu, Jin Xing Lu

**Affiliations:** ^1^ State Key Laboratory of Infectious Disease Prevention and Control, National Institute for Communicable Disease Control and Prevention, Chinese Center for Disease Control and Prevention, Beijing, China; ^2^ Research Center for Micro-Ecological Agent Engineering and Technology of Guangdong Province, Guangzhou, China; ^3^ Collaborative Innovation Center for Diagnosis and Treatment of Infectious Diseases, Hangzhou, China

**Keywords:** *Clostridioides difficile*, cgMLST, epidemiological surveillance, China PIN, evaluation

## Abstract

**Introduction:**

*Clostridioides difficile (C. difficile)* is a nosocomial bacterial pathogen that causes antibiotic-associated diarrhea mediated by cellular exotoxins secreted into the intestine during bacterial growth. Multilocus sequence typing (MLST) and PCR ribotyping are the main molecular typing for *C. difficile*. Whole genome sequencing (WGS) core genome multilocus sequence typing (cgMLST) was developed for genetic evolution and outbreak investigation of *C. difficile* with higher precision and accuracy.

**Methods:**

A total of 699 whole (complete and draft) genome sequences of distinct *C. difficile* strains were used in this study to identify core gene set (2469 core genes) and the cgMLST scheme for the phylogeny analysis of *C. difficile*. This cgMLST pipeline was then carried the Chinese Pathogen Identification Net (China PIN) for surveillance of *C. difficile* in China. Within the China PIN, 195 WGS of *C. difficile* and an outbreak of CDI with 12 WGS of *C. difficile* were used to evaluate the cgMLST pipeline.

**Results:**

The result displayed that mostly tested *C. difficile* isolates could be successfully divided into 5 classic clades and the outbreak event was also successfully identified.

**Discussion:**

The results are meaningful and offer a practicable pipeline for a national-wide surveillance of *C. difficile* in China.

## Introduction

*Clostridioides difficile* is a nosocomial pathogen responsible for gut inflammation, with result of diarrhea or pseudomembranous colitis (Brazier, 2008). *C. difficile* is an anaerobic Gram-positive, spore-forming rod that was first described by the American workers Hall and O’Toole1 who were studying the microbial flora of the meconium and faeces of newborns. Originally, it was named *Bacillus difficilis* owing to the difficulties experienced in culturing it using the technologies available in 1935 (Hall and O'Toole, 1935; Smits et al., 2016). *C. difficile* is a human and animal pathogen causing intestinal infections following disturbance of the gut microbiota, usually as a result of prior antibiotic treatment. *C. difficile* is now widely recognized as the leading cause of nosocomial diarrhea worldwide with associated substantial morbidity and mortality (Peery et al., 2012; Wiegand et al., 2012). Common typing methods for *C. difficile* include PCR ribo-typing, pulsed field gel electrophoresis (PFGE), restriction endonuclease analysis (REA), multi-site sequence typing (MLST), and multi-site variable Tandem Repeat Analysis (MLVA) (Huber et al., 2013). According to the multilocus sequence typing (MLST) scheme established by Griffifiths et al., five distinct phylogenetic lineages (clades 1 to 5) are widely recognized, and an additional clade, clade C-I, was identified, which was confirmed by WGS studies. WGS as a newly developed typing method, provide high-level differentiation between strains and facilitate epidemiological investigations in the short and long term (Lemee et al., 2004; Griffiths et al., 2010; He et al., 2010; Eyre et al., 2013; He et al., 2013; Maiden et al., 2013).

The cgMLST method developed for *C. difficile* was already reported in 2018, which developed a cgMLST target genes from 11 genomes, and further evaluated using 3,025 genomes from GenBank. 2,270 core genes were identified as target genotyping, and the new cgMLST typing scheme was validated with 70 outbreak-related strains (Bletz et al., 2018). In this study, we performed a WGS-based typing using 699 WGS data. From the Whole Genome Multilocus Sequence Typing (wgMLST) we extracted the core gene set and developed the cgMLST scheme for the phylogeny analysis of *C. difficile*. 2649 core genes were identified and retained as cgMLST target genes, and then the cgMLST scheme was used to evaluate the isolates from the outbreak of *C. difficile* infection in the hospital.

In order to improve the laboratory monitoring system for *C. difficile* and share bacterial infectious disease data, we carried the cgMLST protocol established in this study to the China PIN and conducted self-tests on 195 strains of *C. difficile* and 12 strains within an outbreak previously published. The clinical isolation of *C. difficile* is carried out through the visual interface of China PIN to upload data and perform multi-sequence typing analysis. In conclusion, the new cgMLST protocol represents the entire *C. difficile* population, is highly discriminatory in outbreaks, and provides a unique nomenclature that facilitates communication between laboratories. By using the cgMLST analysis solution of *C. difficile* on the platform of the China PIN and using the analysis system and visualization software developed by the pathogen identification network, the molecular typing and outbreak traceability of *C. difficile* can be well carried out.

## Materials and methods

### Whole genome sequences and isolates analyzed in this study

The whole genome sequences of 207 C*. difficile* strains were sequenced in this study (**Table S1**). These strains were isolated from clinical samples in China. A total of 699 WGS of *C. difficile* from the NCBI database (http://www.ncbi.nlm.nih.gov/) were used to screen the cgMLST target genes. The complete genome of *C. difficile* strain 630 (GenBank assembly accession number AM180355.1) was used as the reference genome to determine cgMLST target genes.

All isolates used were cultured on brain heart infusion (BHI) agar plates (Oxoid, UK), supplemented with 5% sheep blood (BaoTe, China) in an anaerobic chamber (80% nitrogen, 10% hydrogen and 10% carbon dioxide) (Mart, NL) at 37°C for 48 h. Typical colonies were picked up and re-cultured on BHI for 24 h before preparation of genomic DNA using the Wizard Genomic DNA Purification Kit (Promega, USA)according to the manufacturer’s instructions.

### Whole-genome sequencing and assembly

All the *C. difficile* strains were sequenced using an Illumina NovaSeq PE150 at the Beijing Novogene Bioinformatics Technology Co., Ltd. In order to ensure the accuracy and reliability of the subsequent information analysis results, the original data must be filtered to obtain valid data (Clean Data) and avoid raw data with low-quality. Raw data was processed in four steps, including removing reads with 5 bp (base pair) of ambiguous bases, removing reads with 20 bp of low quality (≤Q20) bases, adapter contamination, and duplicated reads. Finally, we obtained clean paired-end reads data. Assembly was performed using SOAP denovo v2.04 (Li et al., 2010).

### Definition the target gene of cgMLST

To determine the cgMLST gene set, a genome-wide gene-by-gene comparison was performed using SeqSphere+(Ridom GmbH) within the cgMLST target definer (version 1.4), with parameters of ≥ 90% gene sequence identity and 95% gene sequence overlap. The genomes of *C. difficile* obtaining from the NCBI database were filtered if they met the following criteria: (i) genomes that with contig number ≥200, (ii) genomes that don’t contain all seven MLST genes or with multiple copies (identity ≥90%, overlap = 100%), and (iii) genomes that having <3,000 single copy homologous genes of candidate target genes. Finally, a total of 699 whole genomes were selected, including the *C. difficile* strain 630 as the reference. Certain genes excluded from the cgMLST scheme should meet the following filter parameters: (i) a minimum length filter that discards all genes shorter than 50 bp; (ii) a start codon filter that discards all genes that contain no start codon at the beginning of the gene; (iii) a stop codon filter that discards all genes that contain no stop codon or more than one stop codon or that do not have the stop codon at the end of the gene; (iv) a homologous gene filter that discards all genes with fragments that occur in multiple copies within a genome (with identity of 90% and >100 bp overlap); and (v) a gene overlap filter that discards the shorter gene from the cgMLST scheme if the two genes affected overlap >4 bp. Furthermore, the plasmid and transposon gene filter were performed as followed: (i) filter genes that is highly homologous with *Clostridioides* plasmid genomes (with identity >90%, overlap >95%); (ii) filter genes that is homologous with transposon_db TransposonPSI database (with identity >50%, coverage >70%). The remained genes were then performed in a pairwise comparison within BLAST version 2.2.12, with parameters used as word size 11, mismatch penalty −1, match reward 1, gap open costs 5, and gap extension costs 2 to the query *C. difficile* genomes. All genes of the reference genome that were common in all query genomes with a sequence identity of ≥90% and 100% overlap and, with the default parameter stop codon percentage filter turned on, formed the final cgMLST scheme; this discards all genes that have internal stop codons in > 20% of the query genomes.

### Evaluation of the cgMLST target gene set

To evaluate the cgMLST scheme, firstly, the core genes we screened were used to construct the minimum spanning tree of 207 strains. Secondly, we used the isolates from the outbreak of *C. difficile* infection in the hospital (Jia et al., 2016). Comparison of the agreement of the two clustering methods using single nucleotide polymorphism (SNP) clustering analysis based on our cgMLST protocol for outbreak strains and using WGS-based SNP clustering analysis. For all assembled genome sequences, use the MUMMER software (Version 3.23) to compare with the reference sequence to find all SNPs, merge the SNPs of all genomes into a matrix file according to the position of the reference sequence genome, and filter out the sites containing gaps. As well as SNPs with a distance less than 5, the aligned SNP sequences of all strains are finally obtained, and the best model is automatically selected to construct the evolutionary tree through the iqtree2 software (Version 2.0.6) bootstrap>1000 times.

### cgMLST pipeline carried on China PIN

**Figure 1:**
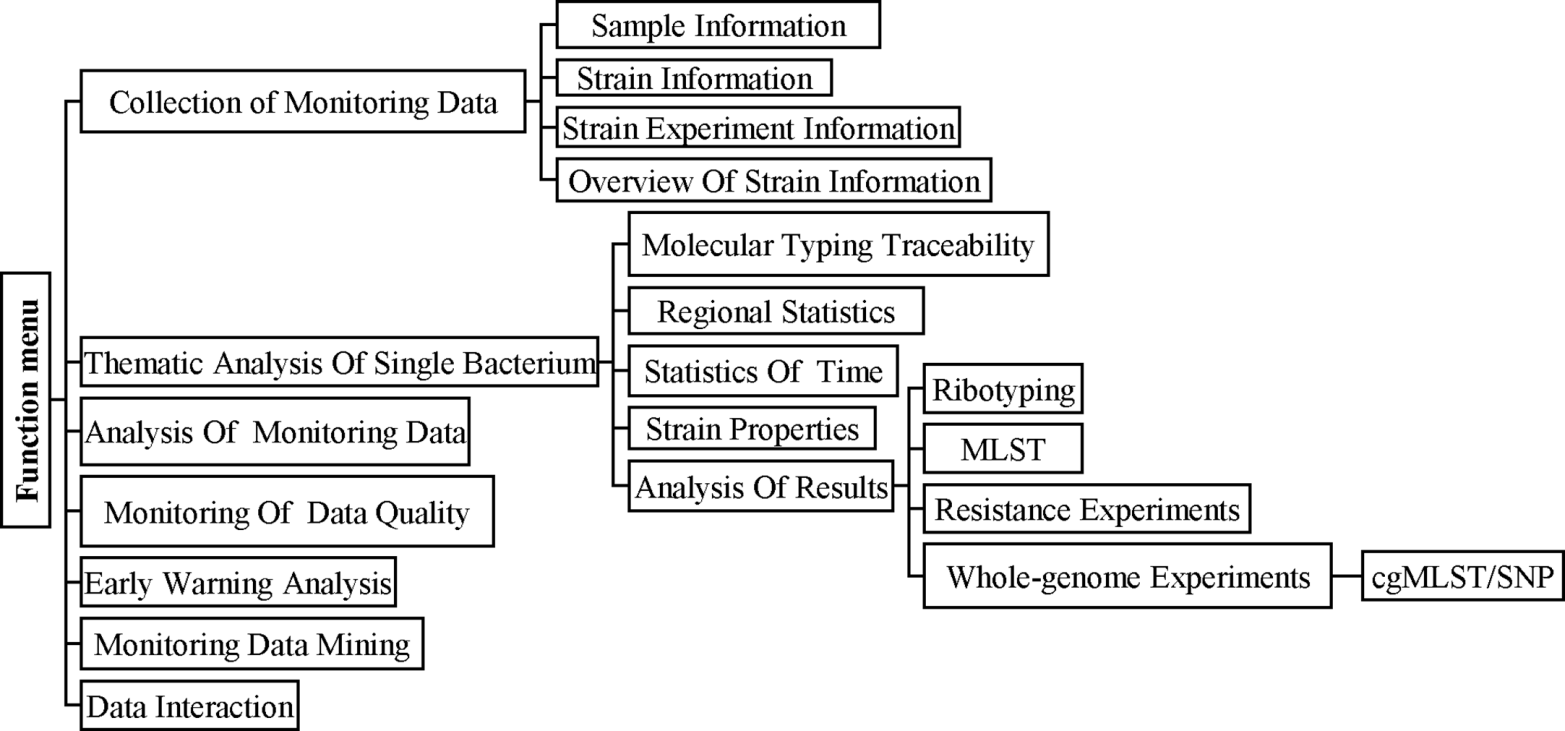
The main functional modules of the China PIN and Operation flowchart. The seven modules in China PIN for surveillance including Collection Of Monitoring Data, Thematic Analysis of Single Bacterium, Analysis of Monitoring Data, Monitoring Of Data Quality, Early Warning Analysis, Monitoring Data Mining, and Data Interaction.

China PIN is based on a networked information platform and adopts new investigation and analysis technologies such as pathogen identification, molecular typing, and genomic epidemiology to carry out the monitoring and prevention of bacterial infectious diseases in China. There are seven modules in China PIN for surveillance including Collection of Monitoring Data, Thematic Analysis of Single Bacterium, Analysis of Monitoring Data, Monitoring of Data Quality, Early Warning Analysis, Monitoring Data Mining, and Data Interaction (**Figure 1**). In order to improve the *C. difficile* laboratory surveillance system and share the surveillance data, the cgMLST pipeline was launched on China PIN. Firstly, we uploaded the demographic data (such as isolation time, location, sample type, sex, age, toxin type, etc.) and genome sequences of the 207 C*. difficile* clinical isolates and another three strains of the ST11 clonal group (21062, 10010, 12038) to China PIN. And then the phylogenetic and molecular analysis of these 210 C*. difficile* isolates were performed according to the cgMLST pipeline carried on China PIN. Subsequently, 22 Sequence Read Archive (SRA) data from (Jia et al., 2016), a retrospective study of the RT027 type outbreak, were downloaded from GenBank and then uploaded to China PIN for further evaluation of the reliability of cgMLST scheme on tracing outbreaks.

### Nucleotide sequence accession number

This Whole Genome Shotgun project has been deposited at GenBank under the Bioproject ID PRJNA704542 with accession number of JAFLER000000000-JAFLEZ000000000, JAFLFA000000000-JAFLFZ000000000, JAFLGA000000000-JAFLGZ000000000, JAFLHA000000000-JAFLHB000000000.

## Results

### Development of the cgMLST scheme

**Figure 2:**
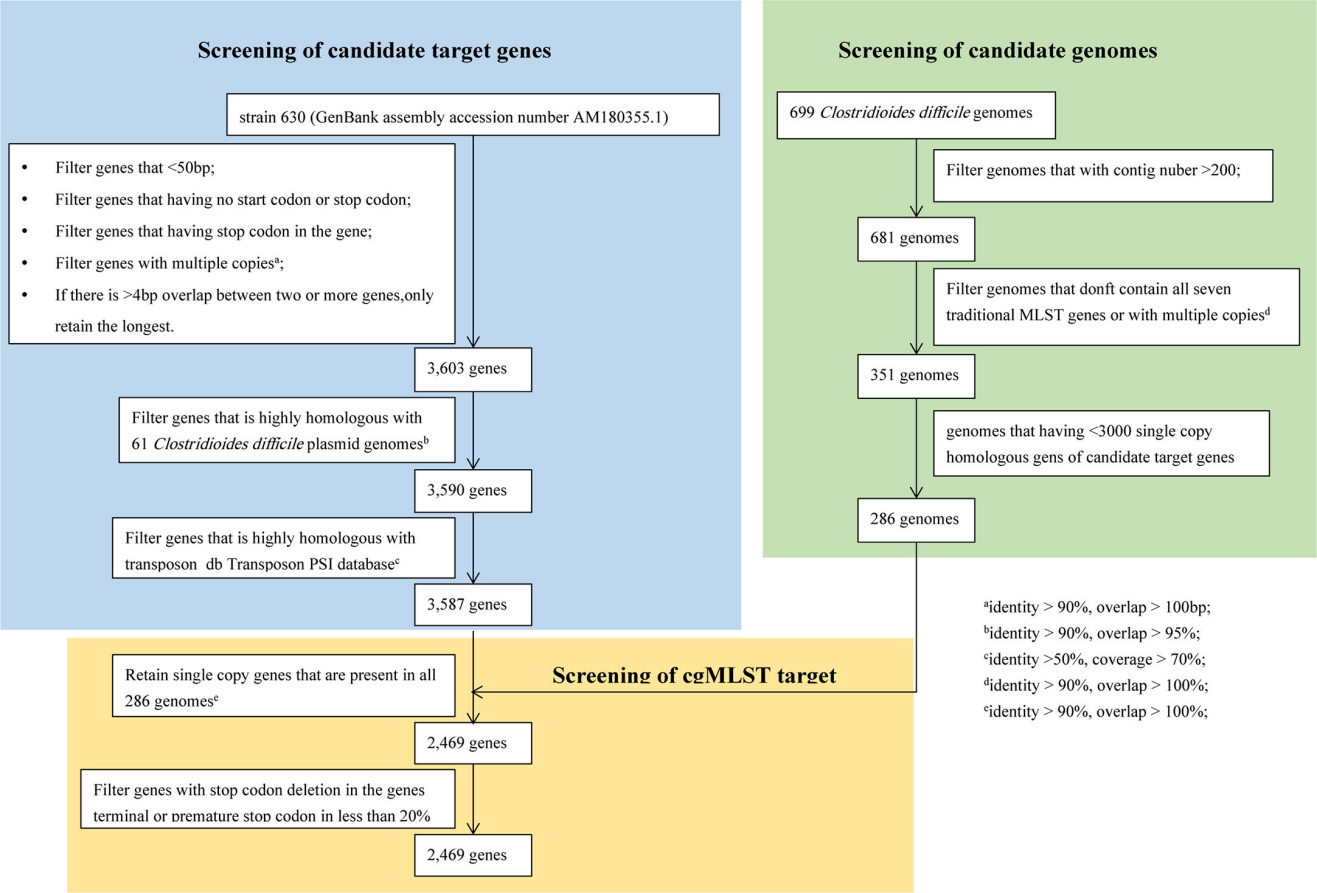
Flowchart to determine cgMLST target genes. There were three steps to determine the final cgMLST target genes. The first step is to select the candidate target genes. The second step is to select the candidate genomes. The last step is to determine the final cgMLST target genes.

It takes three steps to determine the final cgMLST target genes. The first step is to select the candidate target genes. According to the filtering parameters displayed in **Figure 2**, a total of 3587 genes out of 3897 genes was reserved as the candidate target genes using CD 630 (GenBank No. AM180355.1) as the reference genome. The second step is to select the candidate genomes (**Figure 2**). A final of 286 candidate genomes were selected from 699 C*. difficile* genomes downloaded from GenBank following the filtering conditions as below (**Figure 2**): after genomes with number of contigs over 200 were abandoned, 681 genomes were left; then 351 genomes with intact 7 MLST gene loci were kept after removing 330 genomes with incomplete MLST gene loci; finally, genomes with number of single copy genes over 3000 were filtered out. The last step is to determine the final cgMLST target genes. All the 3587 candidate genes should be appeared as single copy in every 286 candidate genomes with identity≥90% and overlap 100%. This led to final 2469 cgMLST target genes even under considering filter of genes deletion of stop codon, or premature stop codon in less than 20%. As shown in **Figure 2**, the developed cgMLST scheme for *C. difficile* included 2,469 target genes, corresponding to 63.36% of the genes with coding DNA sequences (CDS) in the reference genome CD630 (3,897 genes). The core target genes cover 72.56% (3.12 megabases) of the full genome size and were distributed evenly across the genome (**Figure S1**). The average length of the 2,469 target genes was 1264.5 bp (standard deviation [SD], 1022.5 bp; range, 17 to 4,298 bp). A complete list of the core and accessory targets can be found in data set **Table S2**.

### Evaluation of the cgMLST

In order to evaluate the cgMLST scheme, a total of 207 C*. difficile* WGS, which were sequenced in our lab, were analyzed according to the 2469 cgMLST target scheme. The average number of alleles reported for each cgMLST target gene was 36 ± 16 alleles (range, 2 to 120). (**Table S3**). This novel cgMLST scheme was then challenged with different sets of strains (**Table S3**; see also **Table S4**). Out of the genomes of the 207 C*. difficile* strains in our lab, 1,454 to 2,469 cgMLST targets (mean, 90.91%; median, 92.43%) could be extracted. The cgMLST typing results showed that at least 90% of the cgMLST target genes were present in 123 of the 207 genomes examined, with a mean ± SD of 90.91 ± 7.27% of the 2,469 target genes detected per genome for all genomes. In the same light, for the published outbreak (Jia et al., 2016), all isolates contained 2,324 to 2,458 cgMLST targets (mean, 97.70%; median, 98.38%), underlining the representativeness of the cgMLST scheme.

**Figure 3:**
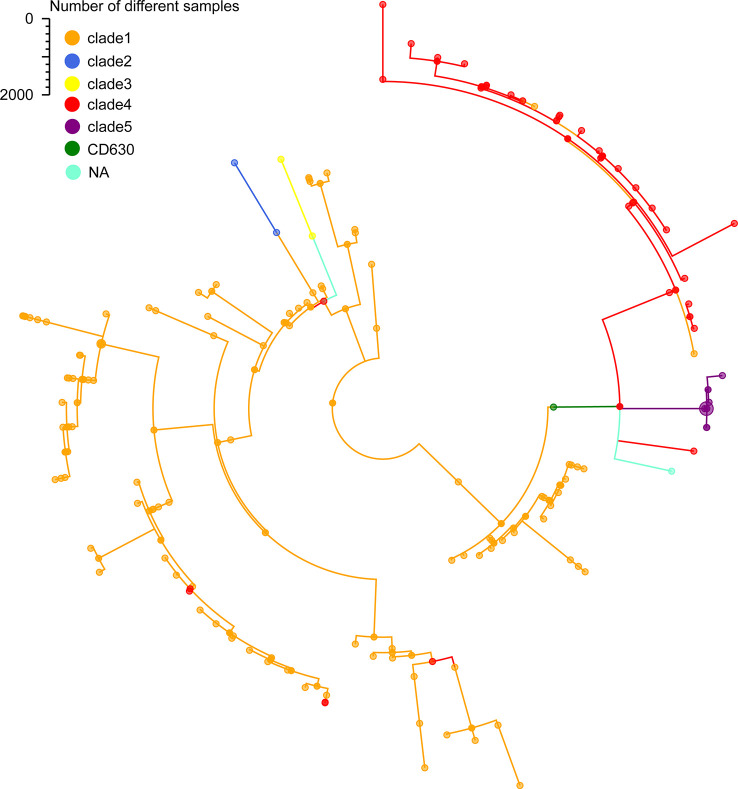
Minimum-spanning tree based on cgMLST allelic profiles of 207 C*. difficile* isolates from our lab. Brown represents the clade 1, blue represents the clade 2, red represents the clade 3, yellow represents the clade 4, purple represents the clade 5, green represents the reference strain 630, light blue represents the NA, not assigned.

To further certain the representativeness of our scheme, we determined 35 STs from the 207 C*. difficile* isolates that were grouped 5 clade using MLST. Only 2 of the 35 STs that were not assigned to any clade, which represented of 1% of the 207 isolates. Additionally, according to the minimum spanning tree of 207 strains using cgMLST, we confirmed 204 distinct core genome sequence types (cgSTs) from the 207 isolates. The minimum spanning tree shows that the 207 C*. difficile* isolates distributed in the whole tree, all the tested isolates were successfully clustered in accordance with their clades except for several strains (**Figure 3**). For example, cgST130, cgST93, cgST87 and cgST18 clustered with clade 1 in the minimum spanning tree based on cgMLST scheme, but these cgSTs grouped within clade4 based upon the MLST scheme (**Figure 3**). Nevertheless, cgST178 and cgST13 clustered with clade 4 in the minimum spanning tree based on cgMLST scheme, but these cgSTs grouped clade 1 based upon the MLST scheme (**Figure 3**). The N1, SN36, SN37 and SN45 isolates shared the identical ST (ST11) based upon the MLST scheme, whereas these ST11 isolates were also separated into same cgSTs(cgST104) using our cgMLST scheme. The result suggests that the four isolates could be from the same outbreak event. And it is illustrated that this cgMLST scheme could clearly distinguish these isolates and demonstrated their phylogenetic relationship.

**Figure 4:**
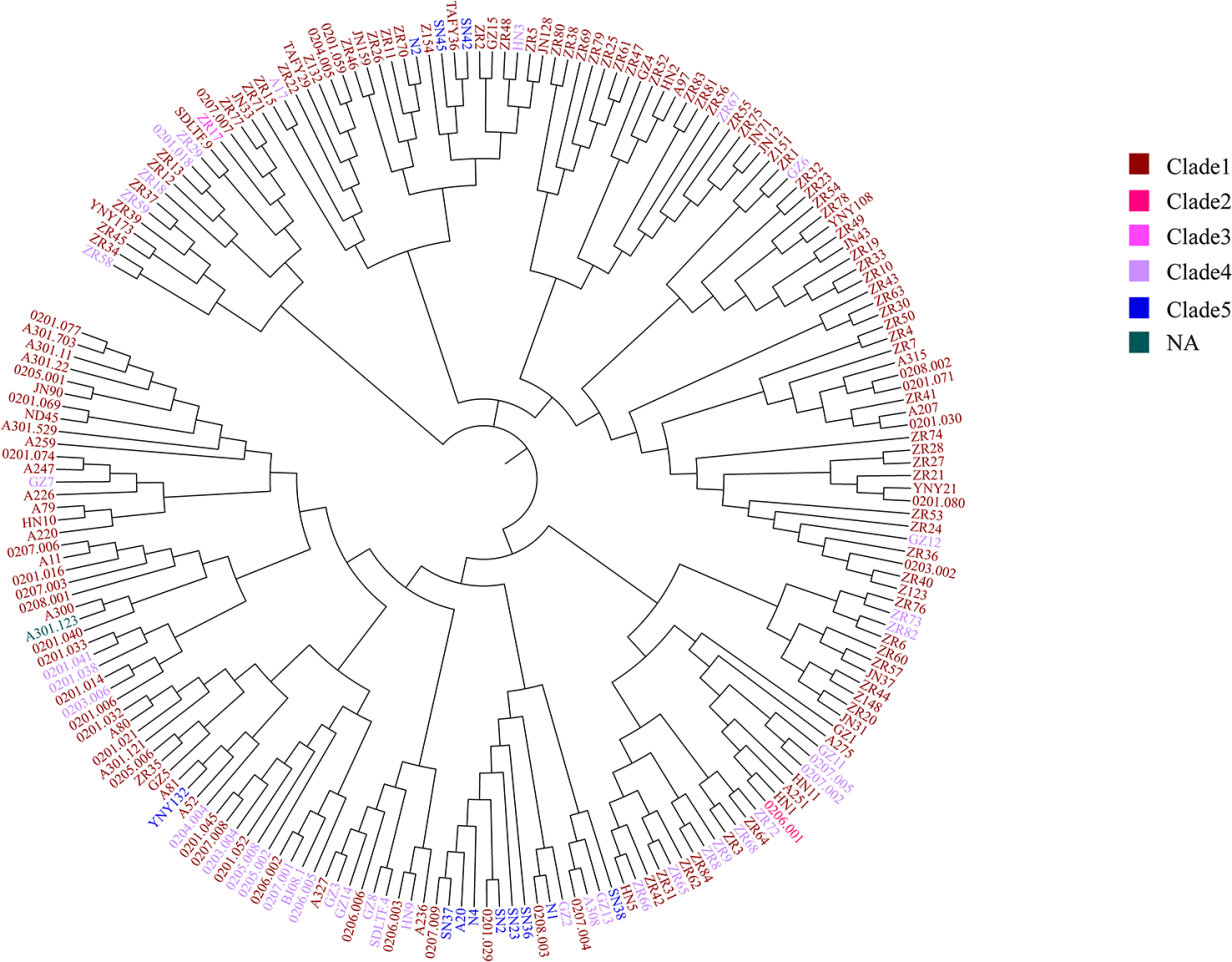
The cgMLST neighbor-joining tree of 207 C*. difficile*. Neighbor joining tree based on *C. difficile* cgMLST allelic profiles determined by the modified chewBACCA software. The dendrogram was created with GrapeTree software version 1.5.0. The branches of the tree were color-coded based on the clade phylogroups indicated on the branches. Brown represents the clade 1, red represents the clade 2, pink represents the clade 3, purple represents the clade 4, blue represents the clade 5, dark green represents the NA, not assigned.

**Figure 5:**
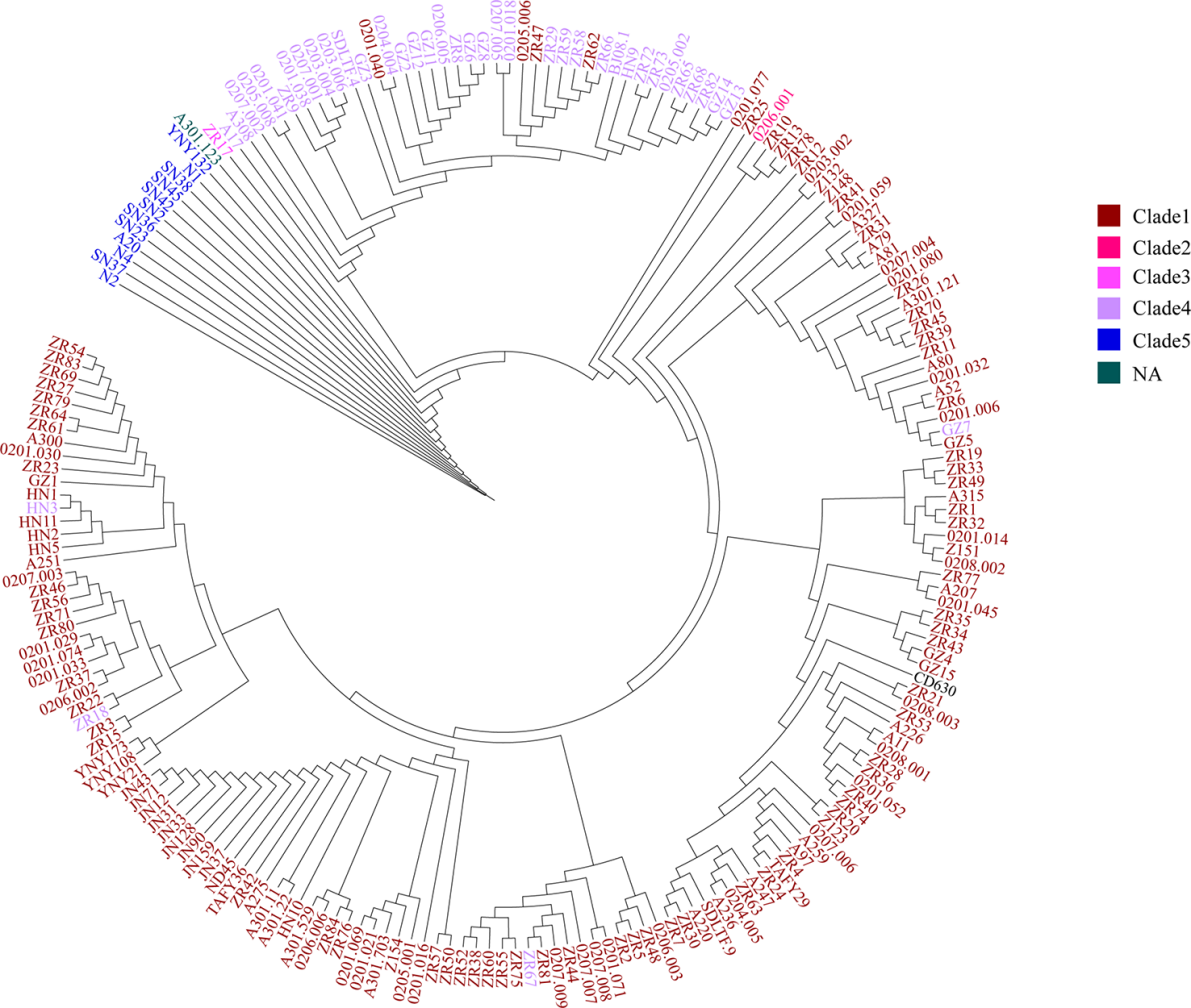
The SNP clustering tree based on cgMLST of 207 C*. difficile*. SNP-based phylogenetic trees were built using PhyML v3.0 under HKY model. Confidence was inferred by running 1,000 bootstrap replicates. The branches of the tree were color-coded based on the clade phylogroups indicated on the branches. The maximum-likelihood (ML) phylogenetic tree of cgSNPs indicating that the clustering of clade 1-5 of 207 isolates demonstrated high genetic relatedness.

To further test the resolution of the newly defined *C. difficile* cgMLST, we made a comparison between cgMLST cluster analysis and core genome SNP-based phylogeny of the 207 C*. difficile* strains. A total of 184,044 variable sites were identified in an alignment concatemer of the 2,469 genes (3.12 Mb). Neighbor joining tree based on *C. difficile* cgMLST allelic profiles, the branches of the tree were color-coded based on the clade phylogroups indicated on the branches (**Figure 4**). The cgMLST neighbor-joining tree (NJ) shows that, most of the strains of clade 1 clustered closely together, however clade 4 and 5 clustering with a more decentralized distribution (**Figure 4**). SNP-based phylogenetic trees (cgSNPs)were built using PhyML v3.0 under HKY model. Confidence was inferred by running 1,000 bootstrap replicates. The maximum-likelihood (ML) phylogenetic tree of cgSNPs indicating that the clustering of clade 1-5 of 207 isolates demonstrated high genetic relatedness (**Figure 5**). For example, in the clade1 cluster, there were 147 out of 151 strains clustered together (97.35%); in the clade4 cluster, there were 36 out of 40 strains clustered together (90%) (**Figure 5**). From the present results, the ML tree of cgSNPs could distinguish between different clades, whereas the NJ tree of cgMLST shows less concordancy with the classical MLST scheme. This does not indicate the in-feasibility of our construction of a core-gene set to distinguish between *C. difficile* categories. The minimum spanning tree based on the core-gene set distinguishes the clade well (**Figure 3**). Different methods of evolutionary tree analysis may result in different tree topologies, and we tend to attribute this difference to the algorithm used to construct the evolutionary tree.

**Figure 6:**
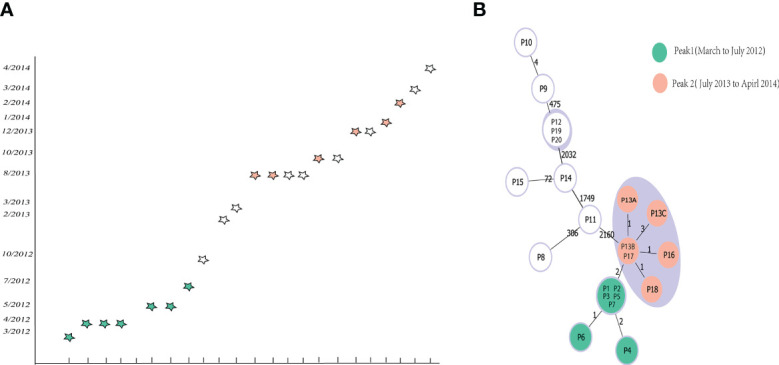
Minimum-spanning tree based on cgMLST target genes and epidemiological curve. The 22 cluster isolates are colored according to their peaks. **(A)** Epidemiological curve. Each five-pointed star represents one isolate, and five-pointed stars are colored according to their peak affiliation. **(B) **Minimum-spanning tree of the reanalyzed sequences based on cgMLST targets. The lines between the circles show the numbers of allelic differences.

Subsequently, we also analyzed the capability of the scheme to differentiate among closely related isolates from an outbreak previously reported from a hospital in China. The WGS of the 22 outbreak isolates were downloaded from Sequence Read Archive (SRA) data (Jia et al., 2016) (**Table S4**), and were used to testify the novel cgMLST scheme. A Minimum Spanning Trees was generated using phyloviz.2.0 under goeBURST full MLST, displaying that using our cgMLST scheme corroborated the previous findings that both peaks were linked together and belonged to the same outbreak clone. In the minimum spanning tree (**Figure 6**), peak 1 clustered together with peak 2, having 2 allelic differences. In peak 1, the isolates (P1, P2, P3, P5 and P7) presented one differing alleles with isolates P6; The isolates P4 presented two differing alleles with isolates P6. In peak 2, the isolates (P13B and P17) presented separately one differing alleles with isolates (P13A, P16 and P18); The isolates (P13B and P17) presented three differing alleles with isolates P13C. Then the isolates P9 presented four differing alleles with isolates P10. Based on these results we finally inferred the threshold, i. e. the maximum number of differing alleles for isolates that are likely to belong to the same clone, as ≤ 6 alleles. So, isolates holding genotypes within this threshold are grouped into the same Cluster Type (CT) (**Figure 6**).

### Application of this novel cgMLST scheme for surveillance on China PIN

**Figure 7:**
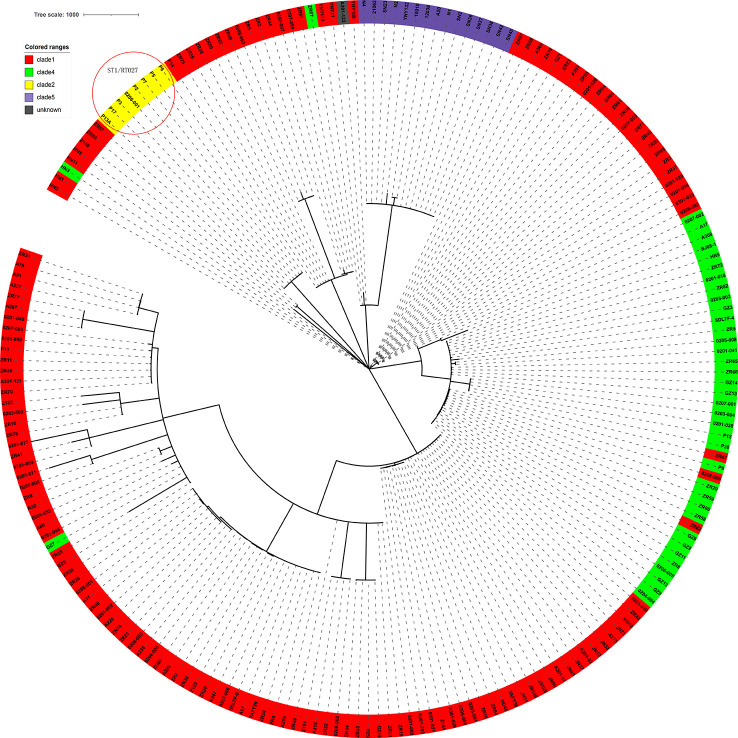
The 210 strains *C. difficile* cgMLST multiple sequence analysis. Clades were added and color-coded. The clades of these strains could be well distinguished according to this cgMLST typing scheme, with only minor individual differences. The red circle in the top left corner shows the retrospective study of the RT027 type outbreak.

The genome sequences of the 207 C*. difficile* clinical isolates, together with another three strains from the ST11 clonal group (21062, 10010, 12038) were uploaded to China PIN. Due to the parameters set on China PIN (Contig ≤ 200, scaffold ≤ 100), 195 strains were finally successfully uploaded. Phylogenetic and molecular analysis of these 195 C. *difficile* isolates were then performed according to this novel cgMLST pipeline carried on China PIN. The results indicated that the clades of these strains could be well distinguished according to this cgMLST typing scheme, with only minor individual differences (**Figure 7**). For example, in the clade1 cluster, there were 148 out of 151 strains clustered together (98.01%); in the clade4 cluster, there were 37 out of 40 strains clustered together (92.5%) (**Figure 7**). Furthermore, 22 SRA data from (Jia et al., 2016) a retrospective study of the RT027 type outbreak, were downloaded from GenBank, and then 12 out of the 22 isolates meeting the parameters (Contig ≤ 200, scaffold ≤ 100) were successfully uploaded to China PIN. The phylogenetic and molecular analysis of these 12 isolates were performed according to the cgMLST pipeline carried on China PIN, which indicated that the outbreak of these strains could be well distinguished (**Figure 7**). It shows that P2, P3, P5, P6, P7, P13A and P17 clustered together, and these seven strains belonging to ST1. P14 clustered with ZR75, which belonged to ST8. P11 clustered with ZR11, which belong to ST2. According to the phylogenetic tree built on the total 210 C*. difficile* isolates using the cgMLST pipeline carried on China PIN (**Figure 7**), all the isolates with ST1/RT027 type including previous outbreak strains and 1 isolate (0206001) from our routine surveillance, clustered together and discriminated clearly from other clusters (**Figure 7**). All the clusters were coincident with classical *C*. *difficile* population structure, clade1-clade 5 (**Figure 7**). The results illustrated that this novel cgMLST pipeline carried on China PIN could work efficiently for identifying outbreaks and genetic analysis, which could be a useful tool for surveillance of *C*. *difficile* in China.

## Discussion

In recent years, WGS has been widely used in the genetic evolution, population migration and epidemiological analysis of pathogenic bacteria (Mutreja et al., 2011; Didelot et al., 2015; Croucher et al., 2013; Holt et al., 2015). At the same time, a core genome MLST (cgMLST) strategy has been used to differentiate between bacterial strains with even greater reliability than traditional MLST approaches, as cgMLST strategies incorporate more sequence data than the 5 to 8 housekeeping genes typically sequenced for MLST (Kimura, 2018; Patino et al., 2018). Among multiple strain typing tools, cgMLST has the highest number of marker sites and the highest density, and it is also more sensitive to subtle variation among strains. Compared with other commonly used typing markers, the high density of cgMLST can extend the resolution of strain typing identification to the clonal level, which is greatly enhanced compared to the species level of 16S rDNA and the clade level of conventional 7-locus MLST. Such cgMLST approaches have been successfully used to type many pathogens, including *Mycoplasma synoviae*, *Brucella* spp., *Yersinia*, *Listeria monocytogenes*, *Staphylococcus capitis, Staphylococcus argenteus*, and *Staphylococcus aureus* (Savin et al., 2019; Ghanem and El-Gazzar, 2018; Sankarasubramanian et al., 2019; Chen et al., 2016; Stenmark et al., 2019; Giske et al., 2019; Chen et al., 2020). WGS is also widely used in *C. difficile* clustering and population structure. In 2018 Bletz et al. (Bletz et al., 2018) proposed the first *C. difficile* cgMLST typing method, using total 11 representative isolates that were included for cgMLST definition, which resulting 2,270 cgMLST genes that were present in all isolates. In the present study, we performed core gene screening using 699 WGS data and finally identified and retained 2649 core genes as cgMLST target genes, and then the cgMLST scheme was used to evaluate *C. difficile* infection outbreak isolates in hospitals. Most importantly, the cgMLST pipeline was launched on China PIN and performed self-testing of *C. difficile* isolates from our laboratory and strains from hospital outbreaks. Data were uploaded through the China PIN visualization interface and subjected to multiple sequence typing analysis. The results illustrated that this novel cgMLST pipeline carried on China PIN could work efficiently for identifying outbreaks and genetic analysis, which could be a useful tool for surveillance of *C*. *difficile* in China.

In this study, we retained 2469 core target genes in our cgMLST scheme, corresponding to 63.36% of the reference genome. The percentage of core genes from the reference genome was 50.4% in 2018 Bletz et al. (Bletz et al., 2018). In prior studies, the proportion of core genes from the reference genome has ranged from 13.3% to 59.33% (Ruppitsch et al., 2015; Zhou et al., 2017; Bialek-Davenet et al., 2014). Differences in the proportion of candidate genomes included in each study influence the number and thus the percentage of core genes. In the MLST scheme, housekeeping genes were evenly spaced throughout the reference genome, as this is critical for such schemes (Do et al., 2010). Similarly, the target genes in this study were distributed throughout the genome (**Figure S1**), confirming the validity and utility of this core gene set.

Then, 286 candidate genomes from the 699 C*. difficile* genomes were finally used for screening cgMLST target genes in current investigation, while in 2018, Bletz et al. used 11 genomes for screening cgMLST target genes. Our study might have more robust data support since the size of our data is significantly larger than that of other studies. A phylogenetic study of 207 C*. difficile* strains recovered in our lab was conducted to confirm the representativeness of our cgMLST scheme. Comparing the cgMLST-NJ tree with the cgSNP-ML tree, it appears from the present results that in the ML tree of cgSNP, strains of different clade origins are better clustered on one branch, while the NJ tree of cgMLST shows a more heterogeneous distribution. It does not indicate the infeasibility of our construction of a core-gene set to distinguish between *C. difficile* categories. Furthermore, the minimum spanning tree based on the core-gene set distinguishes the clade well. Different methods of evolutionary tree analysis may result in different tree topology, and we tend to attribute this difference to the algorithm used to construct the evolutionary tree. The percentage of strains containing core-gene fluctuated from 50% to 99%, partly due to differences in genome assembly and partly due to the strict parameters we set (coverage = 100%, similarity > 90%). The SNP-based tree is less affected by this, and when a fragment is missing a few bp, the comparison region is also missing a few bp, which has less impact on the results of SNP calling as long as the SNP sites are not located at both ends. However, these strict parameters can lead to the absence of this gene during cgMLST typing. NJ tree is a clustering analysis based on the distance matrix of cgMLST between samples. Due to the clustering is based on the distance matrix of cgMLST between samples, and the core gene of a sample is missing, the distance between samples increases or decreases, resulting in clustering results maybe not true to the clade results. The findings demonstrated that our cgMLST scheme could be successfully clustered. Subsequently, we analyzed the ability of the scheme to distinguish closely related strains in outbreak investigations and evaluated the analysis of isolates from *C. difficile* infection outbreaks in hospitals. Based on these results, we finally defined the threshold, in other words, the maximum number of differing alleles for isolates that are likely to belong to the same clone, as ≤ 6 alleles. Isolates sharing genotypes within this threshold are then grouped within the same Cluster Type.

Finally, the advantage of our program is that the cgMLST pipeline launched on the China PIN, which provides an effective technical tool and analysis platform. It can be used to monitor the *C. difficile* infections in China as well as outbreak transmission events. In addition, it could be also used to effectively and rapidly trace or analyze the transmission route. The platform’s surveillance network information system should carry out big data collection and analysis, realize information sharing, implement real-time analysis, and improve the accuracy of epidemic monitoring and analysis. Promote the in-depth integration of epidemiological investigation and analysis of infectious diseases with laboratory surveillance to form a more sensitive and accurate new model of infectious disease surveillance and improve the ability to detect outbreaks and identify their sources. Promote further synergy between CDC and medical institutions to improve the integrated capacity of CDI surveillance and outbreak management, etc. We conducted self-testing of 195 strains of *C. difficile* isolated in our laboratory and 12 strains from hospital outbreaks. The data were uploaded through the China PIN visualization interface and subjected to multiple sequence typing analysis. The results allow for strong molecular typing and outbreak traceability of *C. difficile*.

In summary, this paper successfully established a core gene-based typing method for *C. difficile*, which was subsequently evaluated using the 207 isolates self-tested in our laboratory and data from nosocomial infection outbreaks. Improved traceability and transmission pathway analysis were further performed by carrying the scheme on the platform of the China PIN, using its developed analysis system and visualization software, which demonstrate highly effectiveness and rapid identification of outbreaks of *C. difficile* in China.

## Data availability statement

The datasets presented in this study can be found in online repositories. The names of the repository/repositories and accession number(s) can be found in the article/**Supplementary Material**.

## Author contributions

YYW: Data curation, writing-original, draft preparation. LX: Preparing a graph. WZ: Experimental operation, data collection. WL: Experimental operation, Bacterial culture. XD and GC: Providing assistance for China PIN. LB: Data collection. YW: Paper design guidance, paper revision. JL: Writing-reviewing. All authors contributed to the article and approved the submitted version.
