# Supplementary material for: Application of a core genome sequence typing (cgMLST) pipeline for surveillance of Clostridioides difficile in China
Source: Front Cell Infect Microbiol. 2023 Mar 13;13:1109153. doi: 10.3389/fcimb.2023.1109153 (PMC10040748; doi:10.3389/fcimb.2023.1109153)
Supplement: Supplementary file 1 [file Table_1.docx]

**Table S1.** Characteristics of *C. difficile* strains sequenced in this study

| Strain ID | Isolation origin | | | Molecular subtype | |
| --- | --- | --- | --- | --- | --- |
|  | Isolation location | Isolation date | | MLST type | Clade |
| GZ1 | Guangzhou | | 1980 | 35 | 1 |
| GZ2 | Guangzhou | | 1980 | 37 | 4 |
| GZ3 | Guangzhou | | 1980 | 37 | 4 |
| GZ4 | Guangzhou | | 1980 | 2 | 1 |
| GZ5 | Guangzhou | | 1980 | 2 | 1 |
| GZ6 | Guangzhou | | 1980 | 37 | 4 |
| GZ7 | Guangzhou | | 1980 | 37 | 4 |
| GZ8 | Guangzhou | | 1980 | 37 | 4 |
| GZ11 | Guangzhou | | 1980 | 37 | 4 |
| GZ12 | Guangzhou | | 1980 | 37 | 4 |
| GZ13 | Guangzhou | | 1980 | 37 | 4 |
| GZ14 | Guangzhou | | 1980 | 37 | 4 |
| GZ15 | Guangzhou | | 1980 | 119 | 1 |
| HN1 | Henan | | 2010 | 35 | 1 |
| HN2 | Henan | | 2010 | 35 | 1 |
| HN3 | Henan | | 2010 | 37 | 4 |
| HN5 | Henan | | 2010 | 35 | 1 |
| HN9 | Henan | | 2010 | 37 | 4 |
| HN10 | Henan | | 2010 | 3 | 1 |
| HN11 | Henan | | 2010 | 35 | 1 |
| JN12 | Henan | | 2012 | 3 | 1 |
| JN031 | Henan | | 2012 | 3 | 1 |
| JN033 | Henan | | 2012 | 3 | 1 |
| JN037 | Henan | | 2012 | 3 | 1 |
| JN043 | Henan | | 2012 | 3 | 1 |
| JN071 | Henan | | 2012 | 3 | 1 |
| JN090 | Henan | | 2012 | 3 | 1 |
| JN128 | Henan | | 2012 | 3 | 1 |
| JN159 | Henan | | 2012 | 3 | 1 |
| ZR1 | Beijing | | 2010-2011 | 3 | 1 |
| ZR2 | Beijing | | 2010-2011 | 102 | 1 |
| ZR3 | Beijing | | 2010-2011 | 35 | 1 |
| ZR4 | Beijing | | 2010-2011 | 54 | 1 |
| ZR5 | Beijing | | 2010-2011 | 55 | 1 |
| ZR6 | Beijing | | 2010-2011 | 2 | 1 |
| ZR7 | Beijing | | 2010-2011 | 54 | 1 |
| ZR8 | Beijing | | 2010-2011 | 37 | 4 |
| ZR9 | Beijing | | 2010-2011 | 37 | 4 |
| ZR10 | Beijing | | 2010-2011 | 118 | 1 |
| ZR11 | Beijing | | 2010-2011 | 2 | 1 |
| ZR12 | Beijing | | 2010-2011 | 15 | 1 |
| ZR13 | Beijing | | 2010-2011 | 118 | 1 |
| ZR15 | Beijing | | 2010-2011 | 15 | 1 |
| ZR17 | Beijing | | 2010-2011 | 5 | 3 |
| ZR18 | Beijing | | 2010-2011 | 37 | 4 |
| ZR19 | Beijing | | 2010-2011 | 35 | 1 |
| ZR20 | Beijing | | 2010-2011 | 54 | 1 |
| ZR21 | Beijing | | 2010-2011 | 54 | 1 |
| ZR22 | Beijing | | 2010-2011 | 35 | 1 |
| ZR23 | Beijing | | 2010-2011 | 35 | 1 |
| ZR24 | Beijing | | 2010-2011 | 54 | 1 |
| ZR25 | Beijing | | 2010-2011 | 117 |  |
| ZR26 | Beijing | | 2010-2011 | 2 | 1 |
| ZR27 | Beijing | | 2010-2011 | 99 | 1 |
| ZR28 | Beijing | | 2010-2011 | 29 | 1 |
| ZR29 | Beijing | | 2010-2011 | 37 | 4 |
| ZR30 | Beijing | | 2010-2011 | 54 | 1 |
| ZR31 | Beijing | | 2010-2011 | 54 | 1 |
| ZR32 | Beijing | | 2010-2011 | 3 | 1 |
| ZR33 | Beijing | | 2010-2011 | 100 | 1 |
| ZR34 | Beijing | | 2010-2011 | 48 | 1 |
| ZR35 | Beijing | | 2010-2011 | 48 | 1 |
| ZR36 | Beijing | | 2010-2011 | 54 | 1 |
| ZR37 | Beijing | | 2010-2011 | 35 | 1 |
| ZR38 | Beijing | | 2010-2011 | 8 | 1 |
| ZR39 | Beijing | | 2010-2011 | 2 | 1 |
| ZR40 | Beijing | | 2010-2011 | 54 | 1 |
| ZR41 | Beijing | | 2010-2011 | 92 | 1 |
| ZR42 | Beijing | | 2010-2011 | 3 | 1 |
| ZR43 | Beijing | | 2010-2011 | 48 | 1 |
| ZR44 | Beijing | | 2010-2011 | 8 | 1 |
| ZR45 | Beijing | | 2010-2011 | 3 | 1 |
| ZR46 | Beijing | | 2010-2011 | 35 | 1 |
| ZR47 | Beijing | | 2010-2011 | 28 | 1 |
| ZR48 | Beijing | | 2010-2011 | 55 | 1 |
| ZR49 | Beijing | | 2010-2011 | 100 | 1 |
| ZR50 | Beijing | | 2010-2011 | 53 | 1 |
| ZR52 | Beijing | | 2010-2011 | 8 | 1 |
| ZR53 | Beijing | | 2010-2011 | 54 | 1 |
| ZR54 | Beijing | | 2010-2011 | 35 | 1 |
| ZR55 | Beijing | | 2010-2011 | 8 | 1 |
| ZR56 | Beijing | | 2010-2011 | 35 | 1 |
| ZR57 | Beijing | | 2010-2011 | 53 | 1 |
| ZR58 | Beijing | | 2010-2011 | 37 | 4 |
| ZR59 | Beijing | | 2010-2011 | 37 | 4 |
| ZR60 | Beijing | | 2010-2011 | 8 | 1 |
| ZR61 | Beijing | | 2010-2011 | 118 | 1 |
| ZR62 | Beijing | | 2010-2011 | 55 | 1 |
| ZR63 | Beijing | | 2010-2011 | 34 | 1 |
| ZR64 | Beijing | | 2010-2011 | 35 | 1 |
| ZR65 | Beijing | | 2010-2011 | 37 | 4 |
| ZR66 | Beijing | | 2010-2011 | 37 | 4 |
| ZR67 | Beijing | | 2010-2011 | 37 | 4 |
| ZR68 | Beijing | | 2010-2011 | 37 | 4 |
| ZR69 | Beijing | | 2010-2011 | 35 | 1 |
| ZR70 | Beijing | | 2010-2011 | 2 | 1 |
| ZR71 | Beijing | | 2010-2011 | 35 | 1 |
| ZR72 | Beijing | | 2010-2011 | 37 | 4 |
| ZR73 | Beijing | | 2010-2011 | 37 | 4 |
| ZR74 | Beijing | | 2010-2011 | 54 | 1 |
| ZR75 | Beijing | | 2010-2011 | 8 | 1 |
| ZR76 | Beijing | | 2010-2011 | 8 | 1 |
| ZR77 | Beijing | | 2010-2011 | 129 | 1 |
| ZR78 | Beijing | | 2010-2011 | 118 | 1 |
| ZR79 | Beijing | | 2010-2011 | 35 | 1 |
| ZR80 | Beijing | | 2010-2011 | 35 | 1 |
| ZR81 | Beijing | | 2010-2011 | 8 | 1 |
| ZR82 | Beijing | | 2010-2011 | 37 | 4 |
| ZR83 | Beijing | | 2010-2011 | 35 | 1 |
| ZR84 | Beijing | | 2010-2011 | 3 | 1 |
| BJ08 | Beijing | | 2010-2011 | 37 | 4 |
| 0201-006 | Shanghai | | 2014 | 2 | 1 |
| 0201-014 | Shanghai | | 2014 | 3 | 1 |
| 0201-016 | Shanghai | | 2014 | 35 | 1 |
| 0201-018 | Shanghai | | 2014 | 37 | 4 |
| 0201-021 | Shanghai | | 2014 | 3 | 1 |
| 0201-029 | Shanghai | | 2014 | 35 | 1 |
| 0201-030 | Shanghai | | 2014 | 35 | 1 |
| 0201-032 | Shanghai | | 2014 | 14 | 1 |
| 0201-033 | Shanghai | | 2014 | 35 | 1 |
| 0201-038 | Shanghai | | 2014 | 81 | 4 |
| 0201-040 | Shanghai | | 2014 | 102 | 1 |
| 0201-041 | Shanghai | | 2014 | 37 | 4 |
| 0201-045 | Shanghai | | 2014 | 129 | 1 |
| 0201-052 | Shanghai | | 2014 | 54 | 1 |
| 0201-059 | Shanghai | | 2014 | 102 | 1 |
| 0201-069 | Shanghai | | 2014 | 8 | 1 |
| 0201-071 | Shanghai | | 2014 | 149 | 1 |
| 0201-074 | Shanghai | | 2014 | 35 | 1 |
| 0201-077 | Shanghai | | 2014 | 33 | 1 |
| 0201-080 | Shanghai | | 2014 | 2 | 1 |
| 0203-002 | Shanghai | | 2014 | 34 | 1 |
| 0203-004 | Shanghai | | 2014 | 81 | 4 |
| 0203-006 | Shanghai | | 2014 | 81 | 4 |
| 0204-004 | Xi'an | | 2014 | 37 | 4 |
| 0204-005 | Xi'an | | 2014 | 54 | 1 |
| 0205-001 | Shanghai | | 2014 | 3 | 1 |
| 0205-002 | Shanghai | | 2014 | 37 | 4 |
| 0205-006 | Shanghai | | 2014 | 3 | 1 |
| 0205-008 | Shanghai | | 2014 | 37 | 4 |
| 0206-001 | Hangzhou | | 2014 | 1 | 2 |
| 0206-002 | Hangzhou | | 2014 | 35 | 1 |
| 0206-003 | Hangzhou | | 2014 | 55 | 1 |
| 0206-005 | Hangzhou | | 2014 | 37 | 4 |
| 0206-006 | Hangzhou | | 2014 | 3 | 1 |
| 0207-001 | Beijing | | 2014 | 81 | 4 |
| 0207-002 | Beijing | | 2014 | 332 | 4 |
| 0207-003 | Beijing | | 2014 | 35 | 1 |
| 0207-004 | Beijing | | 2014 | 2 | 1 |
| 0207-005 | Beijing | | 2014 | 37 | 4 |
| 0207-006 | Beijing | | 2014 | 54 | 1 |
| 0207-007 | Beijing | | 2014 | 8 | 1 |
| 0207-008 | Beijing | | 2014 | 42 | 1 |
| 0207-009 | Beijing | | 2014 | 8 | 1 |
| 0208-001 | Guangzhou | | 2014 | 54 | 1 |
| 0208-002 | Guangzhou | | 2014 | 3 | 1 |
| 0208-003 | Guangzhou | | 2014 | 54 | 1 |
| SDLTF-4(TAFY04) | Shandong | | 2013 | 37 | 4 |
| SDLTF-9(TAFY09) | Shandong | | 2013 | 54 | 1 |
| TAFY29 | Shandong | | 2013 | 54 | 1 |
| TAFY36 | Shandong | | 2013 | 3 | 1 |
| A11 | Shandong | | 2018.1 | 54 | 1 |
| A17 | Shandong | | 2017.11 | 124 | 4 |
| A20 | Shandong | | 2017.11 | 11 | 5 |
| A52 | Shandong | | 2017.11 | 2 | 1 |
| A79 | Shandong | | 2018.1 | 102 | 1 |
| A80 | Shandong | | 2018.1 | 2 | 1 |
| A81 | Shandong | | 2018.1 | 102 | 1 |
| A97 | Shandong | | 2017.11 | 54 | 1 |
| A207 | Shandong | | 2018.1 | 129 | 1 |
| A220 | Shandong | | 2017.11 | 54 | 1 |
| A226 | Shandong | | 2017.11 | 54 | 1 |
| A236 | Shandong | | 2018.1 | 54 | 1 |
| A247 | Shandong | | 2018.1 | 54 | 1 |
| A251 | Shandong | | 2018.1 | 35 | 1 |
| A259 | Shandong | | 2017.11 | 54 | 1 |
| A275 | Shandong | | 2017.11 | 3 | 1 |
| A300 | Shandong | | 2017.11 | 35 | 1 |
| A308 | Shandong | | 2017.12 | 254 | 4 |
| A315 | Shandong | | 2017.12 | 3 | 1 |
| A327 | Shandong | | 2017.11 | 102 | 1 |
| A301.11 | Beijing | | 2017.9 | 3 | 1 |
| A301.22 | Beijing | | 2018.8 | 3 | 1 |
| A301.121 | Beijing | | 2018.8 | 2 | 1 |
| A301.123 | Beijing | | 2018.8 | 473 | unknown |
| A301.529 | Beijing | | 2018.8 | 3 | 1 |
| A301.703 | Beijing | | 2018.8 | 3 | 1 |
| N1 | Shandong | | 2017.11 | 11 | 5 |
| N2 | Shandong | | 2017.11 | 11 | 5 |
| N4 | Shangdong | | 2017.11 | 11 | 5 |
| ND45 | Shangdong | | 2017.11 | 3 | 1 |
| SN2 | Shangdong | | 2018.1 | 11 | 5 |
| SN23 | Shangdong | | 2018.1 | 11 | 5 |
| SN36 | Shangdong | | 2018.1 | 11 | 5 |
| SN37 | Shangdong | | 2018.1 | 11 | 5 |
| SN38 | Shangdong | | 2018.1 | 11 | 5 |
| SN42 | Shangdong | | 2018.1 | 11 | 5 |
| SN45 | Shangdong | | 2018.1 | 11 | 5 |
| YNY21 | Yunnan | | 2017.9 | 468 | 1 |
| YNY108 | Yunnan | | 2017.9 | 468 | 1 |
| YNY132 | Yunnan | | 2017.9 | 11 | 5 |
| YNY173 | Yunnan | | 2017.9 | 468 | 1 |
| Z123 | Sichuan | | 2018.1 | 54 | 1 |
| Z132 | Sichuan | | 2018.1 | 29 | 1 |
| Z148 | Sichuan | | 2018.1 | 205 | 1 |
| Z151 | Sichuan | | 2018.1 | 3 | 1 |
| Z154 | Sichuan | | 2018.1 | 3 | 1 |
