# Supplementary material for: Application of a core genome sequence typing (cgMLST) pipeline for surveillance of Clostridioides difficile in China
Source: Front Cell Infect Microbiol. 2023 Mar 13;13:1109153. doi: 10.3389/fcimb.2023.1109153 (PMC10040748; doi:10.3389/fcimb.2023.1109153)
Supplement: Supplementary file 4 [file Table_4.docx]

| Isolate | Clade | MLST ST | PCR ribotype | Toxinotype | NCBI or ENA SRA accession no. (reference) |
| --- | --- | --- | --- | --- | --- |
| M68 | 4 | 37 | 17 | VIII | NC_017175 |
| R20291 | 2 | 17 | 27 | III | NC_013316 |
| P1 | 2 | 1 |  |  | SRX821661 |
| P2 | 2 | 1 |  |  | SRX821763 |
| P3 | 2 | 1 |  |  | SRX821764 |
| P4 | 2 | 1 |  |  | SRX821765 |
| P5 | 2 | 1 |  |  | SRX821766 |
| P6 | 2 | 1 |  |  | SRX821767 |
| P7 | 2 | 1 |  |  | SRX821768 |
| P8 | 1 | 2 |  |  | SRX821769 |
| P9 | 4 | 37 |  |  | SRX821770 |
| P10 | 4 | 37 |  |  | SRX821771 |
| P11 | 1 | 2 |  |  | SRX821772 |
| P12 | 4 | 81 |  |  | SRX821773 |
| P13A | 2 | 1 |  |  | SRX821774 |
| P13B | 2 | 1 |  |  | SRX821775 |
| P13C | 2 | 1 |  |  | SRX821777 |
| P14 | 1 | 8 |  |  | SRX821778 |
| P15 | 1 | 8 |  |  | SRX821779 |
| P16 | 2 | 1 |  |  | SRX821780 |
| P17 | 2 | 1 |  |  | SRX821781 |
| P18 | 2 | 1 |  |  | SRX821782 |
| P19 | 4 | 81 |  |  | SRX821783 |
| P20 | 4 | 81 |  |  | SRX821784 |

**Table S4.** List of 22 C. difficile isolates and genomes (toxinotypes and cluster/outbreak isolates) for evaluation of the novel cgMLST scheme.
